# Geographic distribution and ecological niche of plague in sub-Saharan Africa

**DOI:** 10.1186/1476-072X-7-54

**Published:** 2008-10-23

**Authors:** Simon B Neerinckx, Andrew T Peterson, Hubert Gulinck, Jozef Deckers, Herwig Leirs

**Affiliations:** 1 Evolutionary Ecology Group, Department of Biology, Universiteit Antwerpen, Groenenborgerlaan 171, B-2020 Antwerp, Belgium; 2 Natural History Museum and Biodiversity Research Center, University of Kansas, 1345 Jayhawk Boulevard, Lawrence, KS 66045-7561, USA; 3 Department of Earth and Environmental Sciences, Katholieke Universiteit Leuven, Celestijnenlaan 200 E, B-3001 Heverlee, Belgium; 4 Danish Pest Infestation Laboratory, University of Aarhus, Faculty of Agricultural Sciences, Department of Integrated Pest Management, Skovbrynet 14, DK-2800 Kongens Lyngby, Denmark

## Abstract

**Background:**

Plague is a rapidly progressing, serious illness in humans that is likely to be fatal if not treated. It remains a public health threat, especially in sub-Saharan Africa. In spite of plague's highly focal nature, a thorough ecological understanding of the general distribution pattern of plague across sub-Saharan Africa has not been established to date. In this study, we used human plague data from sub-Saharan Africa for 1970–2007 in an ecological niche modeling framework to explore the potential geographic distribution of plague and its ecological requirements across Africa.

**Results:**

We predict a broad potential distributional area of plague occurrences across sub-Saharan Africa. General tests of model's transferability suggest that our model can anticipate the potential distribution of plague occurrences in Madagascar and northern Africa. However, generality and predictive ability tests using regional subsets of occurrence points demonstrate the models to be unable to predict independent occurrence points outside the training region accurately. Visualizations show plague to occur in diverse landscapes under wide ranges of environmental conditions.

**Conclusion:**

We conclude that the typical focality of plague, observed in sub-Saharan Africa, is not related to fragmented and insular environmental conditions manifested at a coarse continental scale. However, our approach provides a foundation for testing hypotheses concerning focal distribution areas of plague and their links with historical and environmental factors.

## Background

Plague is a rapidly progressing, serious illness in humans that is likely to be fatal if not treated [1,2]. It remains a public health threat in many parts of the world, but particularly in sub-Saharan Africa [3]. Plague is endemic to countries across Africa; however, most human cases are currently being reported from East Africa and Madagascar [3], with > 10 000 cases during the last decade (WHO plague archives, unpublished).

Plague is a zoonotic disease caused by the bacillus *Yersinia pestis*; plague bacteria circulate mainly in rodent hosts and are transmitted between them and to other mammals via adult fleas, and predation or cannibalism, but potentially also by contaminated soil [1,4-8]. The disease is enzootic in a variety of wild rodent species and in diverse habitats [2]. In Africa, plague cases generally occur in seasonal pulses, and show a geographically clearly disjunct distribution in circumscribed foci that are assumed to be correlated with distributions of dominant vectors and rodent reservoirs and their ecology [9]. Many such foci have been identified, and the current observed distribution of human plague appears to coincide with the natural foci; however, a recent World Health Organization report concluded that it is unlikely that all foci have been discovered [7]. In spite of plague's highly focal nature, the ecological understanding of the general distribution pattern of plague across Africa has not been established to date.

Studies of plague in Africa have generally focused at micro-scales, examining host-vector-parasite systems and human social activity patterns within single plague foci [10-14]. These studies can help in identifying the hosts/vectors involved and in evaluating human risk behavior in a particular region, but have been unable to elucidate ecological factors shaping the general pattern of plague's geographic distribution at broader scales. Alternatively, macro-scale studies have been performed to examine the distributional patterns of plague, and potential links with environmental conditions, but not in Africa. In the United States spatial patterns in plague transmission were evaluated in view of changing climates, with the conclusion that observed temporal patterns in plague distributions are consistent with changing climates [15]. In a recent study in the US, the geographic distributions of 13 flea species – potential plague vectors – were predicted and explored [16]. Still in the US, positive relationships were established between human plague incidence, and winter-spring rainfall and elevation [17,18].

In this study, we aim to test the potential of using coarse-resolution environmental factors to predict the geographic distribution of plague across sub-Saharan Africa. Given the observed focal nature of plague, we suspect that environmental factors play a role in the complex plague cycle and so may explain – at least partly – the details of its spatial distribution. To this end, we develop ecological niche models (ENMs) using human plague case occurrence data to explore the potential geographic distribution of plague and its ecological requirements across sub-Saharan Africa. This approach provides a foundation for testing hypotheses concerning focal distributional areas of plague and links with environmental variables. If ecological factors affect the distribution of plague, and these factors can be identified, models can be developed to predict distributions of yet unknown plague foci.

## Results

**Figure 1:**
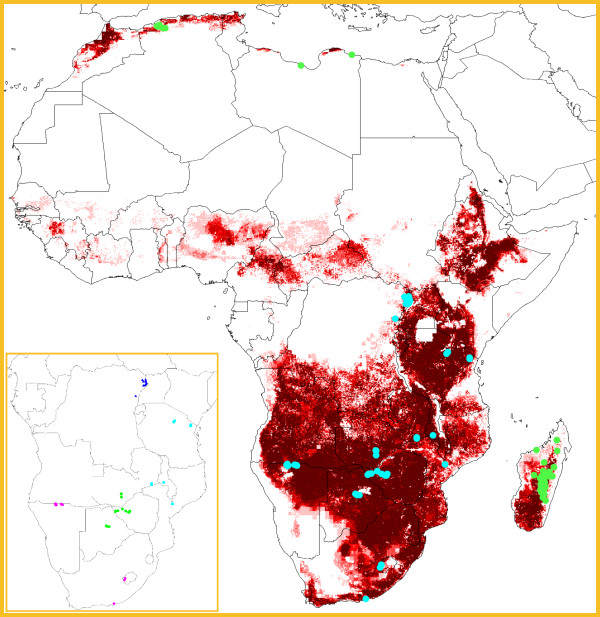
**Geographic overview of plague in Africa**. Inset: 45 occurrence points, colored differently to indicate the four regional subsets: subset A (dark blue triangles); subset B (light blue squares); subset C (green circles); subset D (pink diamonds). Sub-Saharan region shown in inset covers training region used for ENM development. Overall figure: projection of the ecological niche model based on all 45 occurrence locations from Sub-Saharan Africa. Dark shades indicate areas with greater model agreement in predicting areas as suitable for plague. Occurrence points in sub-Saharan Africa on which predictions were based, are shown in light blue; independent test points in Madagascar and North Africa are shown in green.

The plague occurrence data set consists of 45 unique locations from central (Democratic Republic of the Congo and Uganda), eastern (Tanzania, Malawi, and Mozambique), and southern Africa (Botswana, Lesotho, Namibia, South Africa, Zambia, and Zimbabwe; Figure 1). Using the full data set, the overall ENM predicts a broad potential geographic distribution of plague in Africa (Figure 1). The main regions predicted as suitable are south of the Sahara Desert, specifically around Lake Victoria and in the central-southern part of the continent. Several areas where plague has never been reported are also predicted, e.g. regions in Ethiopia, Nigeria, and the Central African Republic. Large areas from sub-Saharan Africa appearing unsuitable for plague are the desert areas (e.g. Kalahari Desert in southern Africa) and the wettest areas, most notably the Congo Basin in the Democratic Republic of the Congo and the coastal regions in eastern and western Africa. Projecting the final model outside the training area (sub-Saharan Africa; see Figure 1), a large part of Madagascar and small areas along the coast of northern Africa are predicted as suitable for plague. Testing the overall model's transferability using independent occurrence data from Madagascar and northern Africa (not used for testing the model) indicates that our model has significantly higher agreement between the niche projection and independent test occurrence data than is expected by chance (both binomial tests, P < 0.001), suggesting that our overall ENM can anticipate the potential distribution of plague occurrences outside the sample area robustly.

**Figure 2:**
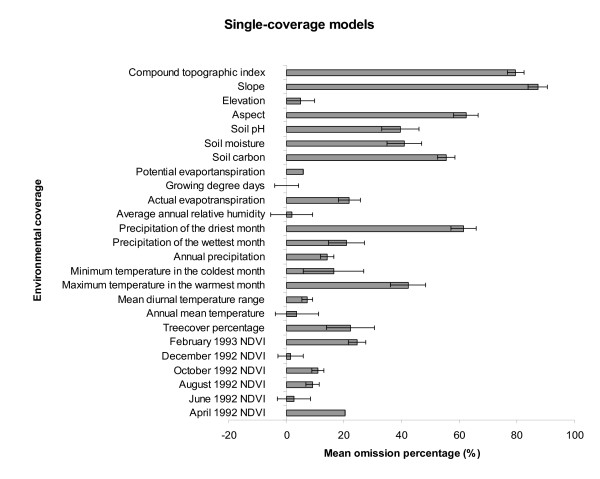
**Relative contributions of single-data environmental data sets**. Horizontal bar graph presents a summary of results of single-data coverage model analyses, indicating mean omission percentages (and standard deviations) calculated based on predictions of 10 best-subset models and independent testing points. Note that positive contribution by the variable is indicated by low values in the single-coverage models.

**Figure 3:**
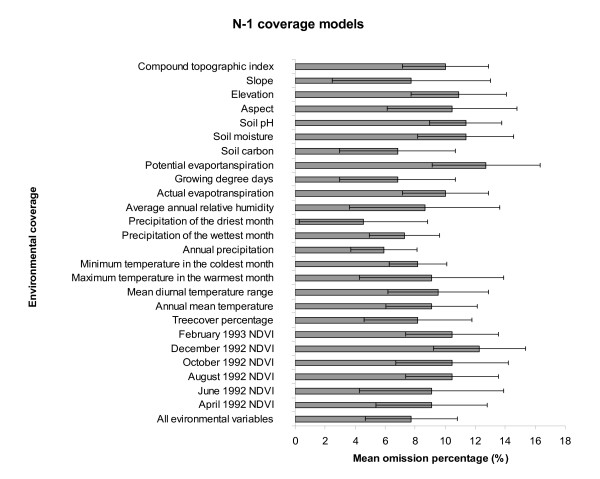
**Relative contributions of N-1 environmental data sets**. Horizontal bar graph presents a summary of results of N-1 coverage model analyses, indicating mean omission percentages (and standard deviations) calculated based on predictions of 10 best-subset models and independent testing points. Note that positive contribution by the variable is indicated by high values in the N-1 coverage models. At the bottom of the bar graph, mean omission percentage (and standard deviation) based on all environmental coverages is presented.

Relative contributions of the various environmental data sets to the overall niche model of plague occurrence were assessed using a jackknife manipulation. All environmental coverages appeared to contribute to the overall model (Figure 2 &3). Precipitation of the driest month contributed the least, while elevation, potential evapotranspiration, mean diurnal temperature range, annual rainfall, and December NDVI appeared to be key factors having substantial influence on the model.

**Figure 4:**
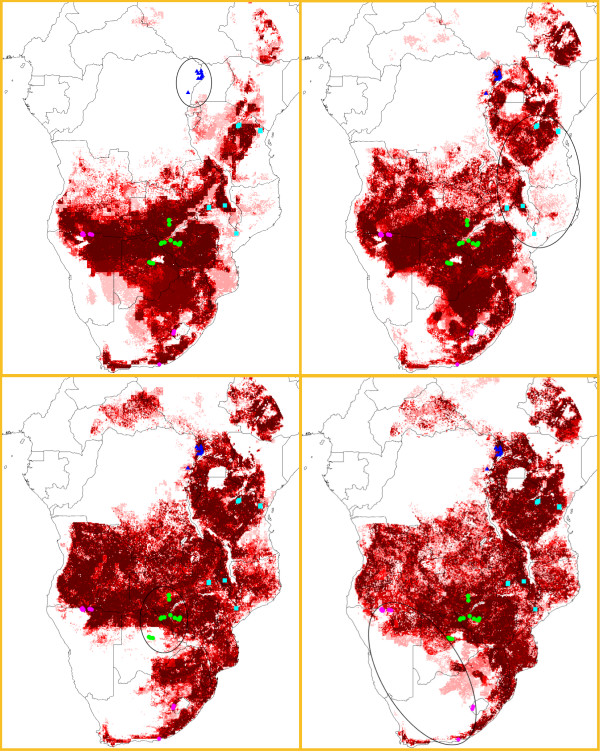
**Potential plague distribution predictions based on regional subsets**. Potential plague distribution predictions were based on all environmental coverages and 3 regional subsets of occurrence points in sub-Saharan Africa. Independent test points from fourth subset are surrounded by a black oval. Dark shades indicate areas with greater model agreement in predicting area as suitable for plague; upper left, subsets B, C and D predict A; upper right, subsets A, C and D predict B; bottom left, subsets A, B and D predict C; and bottom right, subsets A, B, C predict D.

To examine the generality and predictive ability of the ENMs, we developed models based on regional subsets of occurrences. In first tests, with 3-region models predicting the spatial distribution of occurrences in the fourth, model predictivity was poor (Figure 4). For example, the ENM based on subsets B, C, and D predicted 0 of 13 A points successfully; the ENM based on A, C, and D predicted 4 of 9 B points; the ENM based on A, B, and D predicted 9 of 14 C points; and the ENM based on A, B, and C predicted only 2 of 11 D points (Table 1). The ENM based on A, B, and D was the only model for which predictions of the independent occurrence points are statistically better than random (binomial test, P < 0.02). These results thus show that the ENMs in this study based on regional subsets were generally unable to predict independent occurrence points accurately, and that plague may occur in diverse ecological situations across Africa.

**Table 1:** Summary of results of regional jackknife analyses.

	Proportional area predicted present	Total number of test points	Number of test points correctly predicted	P-value
BCD predict A	0.249781	11	0	0.957629
ACD predict B	0.478807	9	4	0.447970
ABD predict C	0.392830	14	9	0.015206
ABC predict D	0.351357	11	2	0.802481

Table presents for every regional subset prediction proportional area predicted present, total number of test points, number of test points correctly predicted, and P-value. Presence was defined as when 5 of 10 of the replicate models predicted presence.

**Figure 5:**
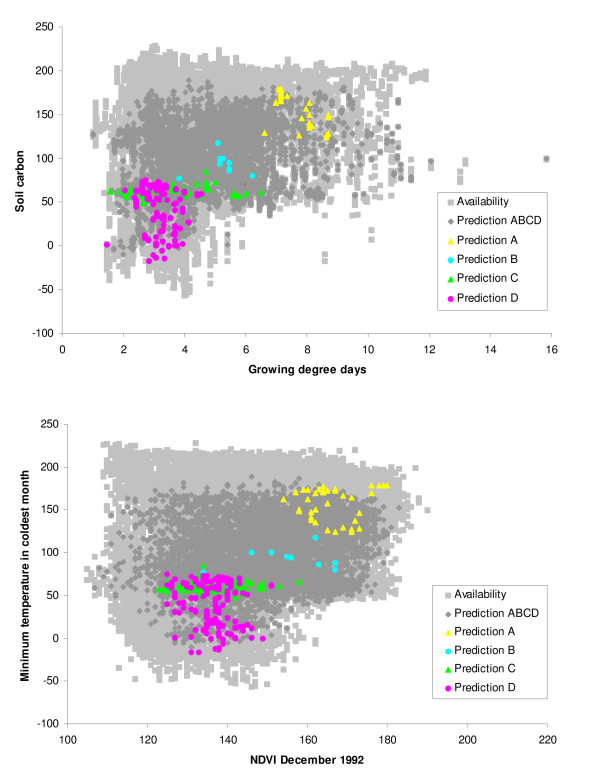
**Visualizations of plague ecological niches**. Examples of visualizations of modeled plague ecological niches are shown in two-dimensional spaces. Shown are all available habitat in the area of observation (light grey squares); general plague niche predicted by all available occurrence points (dark grey diamonds); niche predicted by A (yellow triangles); niche predicted by B (blue circles); niche predicted by C (green triangles); niche predicted by D (pink circles).

To visualize sub-Saharan African plague niches in ecological dimensions, ENM predictions were related to conditions across the landscape. To this end, we integrated four ENMs based on individual regional subsets and all environmental layers with the base environmental layers. Figure 5 presents example visualizations in two dimensions (temperature and soil carbon) for regional subsets to illustrate broad trends: the regional ecological niches are included within a broader, composite ecological niche that is discernible in ENMs based on all available occurrences. This result is consistent with the results of our spatially stratified testing procedure, in which ENMs could not generally predict plague presences in regions outside the training area. More generally, predicted areas based on all available occurrence locations coincide more or less with the diversity of conditions across sub-Saharan Africa; only regions where extreme ecological conditions are present were left out (e.g. the Kalahari Desert in South Africa). In sum, our plague ENMs suggest that sub-Saharan African plague occurs in ecologically diverse landscapes under wide ranges of environmental conditions.

## Discussion

Since 1970, plague has been reported from several African countries, including (in decreasing order of importance) the Democratic Republic of the Congo, Madagascar, Tanzania, Uganda, Angola, Zambia, Mozambique, Zimbabwe, Malawi, South Africa, Lesotho, Botswana, Kenya, Namibia, Algeria, and Libya [7]. Countries presently affected most seriously are the Democratic Republic of the Congo, Madagascar, Tanzania, Uganda, and Mozambique [3]. The overall ENM, which predicted much of sub-Saharan Africa and Madagascar as suitable for plague, seems consistent with these observations: plague has a very broad geographic potential in Africa. It is worth noting that, despite the high number of human plague recordings, only small parts of the Democratic Republic of the Congo were predicted; but indeed, the highly infected northeastern region where plague mostly occurred, i.e. the Ituri district, was accurately predicted by our models. Moreover, in North Africa, our ENMs have significant predictive power, including in northern Algeria where plague reappeared in 2003 after a quiescent period of >50 yr [19,20]. Nonetheless, in spite of statistical significance, we observed some predictive failures as well: areas in the Democratic Republic of the Congo with historical plague occurrences were predicted only at low levels, while some plague-positive areas in Libya were not modeled as suitable.

The broad modeled potential distribution, however, contrasts with the focal nature of plague in Africa: generally, active plague foci are circumscribed, down to areas of just a few hundred square kilometers [21]. We suspected ecological variables as climate, topography, and land cover to play a role in the distribution of plague in Africa – Soviet scientists were the first to bring attention to the relationship between landscapes and the distribution and occurrences of diseases, as plague [1]. Land cover might influence rodent and flea diversity or densities, as climate dynamics showed to influence the abundance of mammals and fleas in the United States and Vietnam, affecting human plague incidence [17,22]. Additionally, recent studies have established relationships between elevation and human plague occurrence [14,18]. From our African analyses, however, it becomes clear that plague can persist in various biotopes under diverse ecological conditions. ENMs based on regional subsets of occurrence points suggest that various ecological subniches may correspond to each plague focus, within a broad niche in which plague seems able to persist. Hence, on this coarse spatial scale, plague occurrences are only predictable in a very general sense. More particularly, persistence of plague in biotopes ranging from dry lowlands to wet highland areas restricts the possibilities of the ENM approach in identifying specific sets of conditions.

The great diversity of ecological circumstances under which plague persists in Africa might be explained by plague ecology being diverse relative to host ecology. Various rodent species are apparently involved in the ecology and epidemiology of plague, and the reservoir(s) in many African plague foci remain(s) unknown [1,6]. It has been suggested that plague survives through a complex suite of rodent species in some areas (e.g., *Mastomys natalensis, Arvicanthis abysinnicus, Lemniscomys striatus, Mus minutoides*, and *Rattus rattus *in the Ituri focus in the Democratic Republic of the Congo), while in others, one species is the main reservoir (e.g., *Rattus rattus *in parts of Madagascar [23]).

A question that arises is what makes a plague focus exist. In our analyses, all environmental variables appear to contribute positively to the overall ENMs, with elevation, potential evapotranspiration, mean diurnal temperature range, annual rainfall, and December NDVI as the most important factors, suggesting that different environmental conditions are likely to influence plague distribution. Yet, uncertainty still exists as to whether absence of plague in rodent populations near an active plague focus is the result of a deterministic process (i.e., the host is present but the conditions are not conducive for plague transmission), or whether it is the result of historical stochasticity (i.e., by chance, plague did not establish in these wild rodent populations or never even reached them). Put another way, the focal plague distribution might result from plague being present only where it was introduced and established locally but without subsequent broad spread from the point of introduction (introduction-driven but dispersal-limited). Alternatively, its spatial distribution may prove broader than observations have suggested, but plague has not been detected or reported in spite of local presence (detection-driven). Apparent lack of plague in an area might also be due to the fact that the region lacks a suitable bridging vector (flea species) to result in transmission to humans (biotically limited).

Certainly, involvement of several species (one or more reservoirs, vectors, incidental hosts, and the pathogen itself), results in a complex system difficult to comprehend. Niche modeling tools may prove valuable, for example, in identifying likely candidate species participating in the transmission cycle [24-26]. Hence, further research using the same tools, but on finer scales, focusing within a single endemic plague region and its surroundings, is needed to explore the mechanisms of a plague focus in greater depth.

It is important to emphasize that our models are based on incidences of human plague derived from historical reports (1970–2007). We are aware that models based on human cases have limitations regarding disease occurrences in natural environments [27]. Underestimation of plague's geographic distribution might be possible; however, since no occurrence data exist for plague in African animals, human records were our only resource. Additionally, available geographic information on occurrences of human plague can be rather coarse in resolution, as plague occurs chiefly in remote areas, often in developing countries. Given incomplete gazetteers, geographic complexities, changing toponyms, or deficient information, we could only assign geographic coordinates to 45 spatially unique occurrence locations, so the occurrence data are not extensive and may not detect phenomena occurring at scales finer than the 5–10 km error margin in georeferencing. Also, since plague occurrences were considered only once, with no weighting to account for multiple cases occurring at single locations, our models do not distinguish epidemic occurrences from ongoing transmission. Finally, it must be remarked that, although ENMs can be developed using relatively small samples of occurrence points [28], sample sizes from the regional subsets of occurrence locations in this study were approaching minimal, such that single data points could change the overall results. However, we developed these ENMs to visualize the ecological distribution of plague in sub-Saharan Africa in ecological space, and not so much to produce accurate predictive maps of plague.

## Conclusion

In this study, an ENM approach was applied to the distribution of plague in sub-Saharan Africa, to identify ecological factors related to the occurrence of the disease. Our main conclusion is that plague in Africa persists in ecologically diverse biotopes, which implies that the typical focality of plague, which is observed here, is not related to fragmented or insular environmental conditions manifested at this coarse scale. Although our overall ENMs may not predict real incidences of human plague accurately, they do outline overall geographic potential, and finer-scale analyses are under development (Neerinckx et al. in preparation).

## Methods

### Human plague occurrence data

Locations of known plague occurrences in endemic regions of sub-Saharan Africa were compiled through an extensive literature search. We first searched the international databases PubMed [29] and Web of Science [30]. Information was derived from scientific publications, supplemented with data from other literature and web-accessible documents. Furthermore, local plague experts, ministries of health in endemic African countries, and the World Health Organization were contacted for additional plague occurrence information. For this study, an occurrence point was defined as a site from which a human bubonic plague case or outbreak of local origin was detected and reported. We assigned geographic coordinates to occurrence locations using world gazetteer databases [31-33] and hardcopy maps. In all, 45 locations from sub-Sahara Africa were georeferenced with a spatial precision of 5–10 km (~0.05–0.1°). More occurrence locations were available, but coordinates could not be assigned for lack of detailed geographic information.

### Environmental data

Environmental data sets (25 'coverages') for ENM development were drawn from five sources. (1) Climatic data layers in the form of seven 'bioclimatic variables' (native resolution 1 × 1 km) were drawn from the WorldClim data set [34], summarizing annual mean temperature, mean diurnal temperature range, maximum temperature of the warmest month, minimum temperature of the coldest month, annual precipitation, and precipitation of the wettest and driest months. (2) Topographic data (native resolution 1 × 1 km) summarizing elevation, aspect, slope, and compound topographic index (a measure of tendency to pool water) were obtained from the U.S. Geological Survey Hydro-1K data set [35]. (3) Seven variables describing soils and ecosystems (native resolution ~10 × 10 km), including actual evapotranspiration, potential evapotranspiration, growing degree days, soil organic carbon, soil moisture, average annual relative humidity, and soil pH were drawn from the Atlas of the Biosphere [36]. (4) One coverage (tree cover percentage) was derived from the Global Land Cover 2000 Project of the European Commission (native resolution 1 × 1 km) [37]. Finally, (5) six monthly maximum NDVI values from the Advanced Very High Resolution Radiometer (AVHRR) sensor for April, June, August, October, and December 1992, and February 1993, were drawn from the University of Maryland Global Land Cover Facility, (native resolution 1 × 1 km) [38]. All data layers were projected in geographic coordinates and generalized to a pixel resolution of ~10 × 10 km for analysis.

### Ecological niche modeling

Our approach to ENM is based on ecological niches defined as the set of environmental conditions under which a species is able to maintain populations without immigration [39,40]. Known occurrences of species were related to digital GIS data layers summarizing environmental variation to develop a quantitative picture of the ecological distribution of the species [39,41]. We used the Genetic Algorithm for Rule-set Prediction (GARP) for ENM development; GARP uses an evolutionary-computing genetic algorithm that develops a set of conditional rules to relate observed occurrences of species to environmental characteristics across the overall study area [42]. Although early evaluations indicated poor predictive ability by GARP [43], more recent analyses with altered performance tests indicate considerable better performance [44,45]. As such, given that GARP has been the basis for essentially all previous ENM analyses of disease systems, the choice for this package was obvious. All modeling in this study was carried out on a desktop implementation of GARP (DesktopGarp) [46].

Within GARP processing, available occurrence data are subdivided as follows: 50% of occurrence points set aside for filtering among replicate models based on their error statistics (extrinsic testing data, see below), 25% used for developing rules (training data), and 25% used for model refinement internal to GARP (intrinsic testing data). Distributional data are converted to raster layers, and 1250 'pseudoabsence' points created by random sampling from areas lacking known presences. GARP works in an iterative process of rule selection, evaluation, testing, and incorporation or rejection. Initially, a method is chosen from a set of 4 basic rule types (atomic rules, bioclimatic rules, range rules, and logistic regression), each a different method for predicting presence versus absence across landscapes [42]. Specific operators designed to mimic chromosomal evolution (e.g., crossing-over among rules, point mutations, deletions, etc.) are then used to modify the initial rules. After each modification, the quality of the rule is evaluated (to maximize both significance and predictive accuracy), based on the intrinsic testing data, and a size-limited set of rules is retained. Because rules are tested based on independent data (intrinsic testing data), performance values reflect the expected performance of the rule, an independent verification that gives a more reliable estimate of true rule performance. The final result is a set of conditional rules that have "evolved" (hence the "genetic" algorithm) for maximum significance and predictive ability; these rules are projected onto the broader landscape to identify a potential geographic distribution for the species [42].

To optimize model performance, we developed 100 replicate models based on independent random subsamples from available occurrences. A "best subset" of 10 models was chosen on the basis of omission (leaving out true potential distributional areas) and commission (including areas not potentially suitable) error statistics calculated based on the extrinsic testing data [47]. Specifically, we used a relative omission threshold, in which the 20% of the models with lowest omission rates were retained. We then chose the 10 models having intermediate levels of commission, i.e., the central 50% of the commission index distribution among the 20 low-omission models. The 10 models selected by this procedure were summed pixel by pixel in ArcView 3.2 to produce a final prediction.

### ENM validation

To test the ability of the ENM algorithm to predict accurately across broad unsampled areas, a "regional jackknife" procedure was used. We divided the overall sub-Saharan Africa data set into four regional subsets to provide a quantitative assessment of transferability (i.e., ability to predict into unsampled areas) of our ecological niche models [44]. Specifically, we focused on spatial stratification (i.e., rather than random splits) of available occurrence data to avoid problems with spatial autocorrelation and consequent non-independence of training and testing data – we used natural gaps as a first criterion (e.g., separating area including Congo and Uganda cases from remaining areas based on an apparent broad disjunction), and then split these disjunct areas further to produce testing regions of approximately even sample sizes (A: 11; B: 9; C: 14; and D: 11). These areas are explicitly arbitrary in nature, but are simply designed to permit testing of the constancy of the niche 'signal' across broad spatial realms. Models were trained based on the available occurrences in three areas, and tested using the distribution of occurrences in the fourth. Predicted presence was defined as areas predicted present by ≥ 5 of 10 of the replicate models. Cumulative binomial probabilities were used to assess the degree to which observed levels of agreement exceeded expectations under the null hypothesis of no association [47], which avoids the low expected frequency limitations of chi-squared testing approaches.

To evaluate the potential geographic distribution of plague across Africa, and to test model transferability, we projected an ENM based on all occurrences onto landscapes covering all of Africa and Madagascar. An independent set of occurrence locations (i.e., not used in model training) from Madagascar (29 points) and northern Africa (10 points), obtained similarly as described above, both with spatial accuracies of 1 km (~0.01°), was used to test the transferability of the final overall model, using methods similar to the binomial testing method described above.

To assess empirical contributions of particular environmental dimensions to the final model, environmental data were manipulated using a jackknife procedure [48]. We developed 25 models each using different combinations of 24 of the 25 environmental coverages; similarly, each coverage was included systematically in single-coverage analyses to evaluate explanatory of each on its own. Inspecting patterns of model performance based on single coverages and based on all other coverages in relation to omission error rates (given that commission "error" includes both true error and apparent error resulting from distributional disequilibrium [39]), then provides a sort of sensitivity analysis, in which we assess the contribution of each coverage to model predictivity [48,49].

### Characterization and comparison of ecological niches

To characterize ecological conditions under which plague exists in different regional foci, we combined GARP predictions based on all occurrences for each regional subset with the original environmental data layers (25 dimensions) into composite grids in ArcGIS 9.1. The attributes tables associated with these grids are effectively lists of all unique environmental combinations across the landscape with associated model predictions (0–10 models predicting presence). To visualize patterns of niche variation, these tables were exported and used to develop scatterplots.

## Competing interests

The authors declare that they have no competing interests.

## Authors' contributions

All authors have read and approved the final manuscript. SN and ATP conceived and designed the study, and drafted the manuscript. HL participated in the study design, helped to draft the manuscript and had supervision over the entire study. JD and HG participated in manuscript revision.
